# Specific Targeting of the Basolateral Amygdala to Projectionally Defined Pyramidal Neurons in Prelimbic and Infralimbic Cortex^1^,^2^,^3^

**DOI:** 10.1523/ENEURO.0002-16.2016

**Published:** 2016-03-21

**Authors:** John Cheriyan, Mahesh K. Kaushik, Ashley N. Ferreira, Patrick L. Sheets

**Affiliations:** 1 Department of Pharmacology and Toxicology, Indiana University School of Medicine-South Bend, South Bend, Indiana 46617; 2 Department of Biological Sciences, University of Notre Dame, Notre Dame, Indiana 46556

**Keywords:** basolateral amygdala, infralimbic, long-range connectivity, medial prefrontal cortex, periaqueductal gray, prelimbic

## Abstract

Adjacent prelimbic (PL) and infralimbic (IL) regions in the medial prefrontal cortex have distinct roles in emotional learning. A complete mechanistic understanding underlying this dichotomy remains unclear. Here we explored targeting of specific PL and IL neurons by the basolateral amygdala (BLA), a limbic structure pivotal in pain and fear processing. In mice, we used retrograde labeling, brain-slice recordings, and adenoviral optogenetics to dissect connectivity of ascending BLA input onto PL and IL neurons projecting to the periaqueductal gray (PAG) or the amygdala. We found differential targeting of BLA projections to PL and IL cortex. Activating BLA projections evoked excitatory and inhibitory responses in cortico-PAG (CP) neurons in layer 5 (L5) of both PL and IL cortex. However, all inhibitory responses were polysynaptic and monosynaptic BLA input was stronger to CP neurons in IL cortex. Conversely, the BLA preferentially targeted corticoamygdalar (CA) neurons in layer 2 (L2) of PL over IL cortex. We also reveal that BLA input is projection specific by showing preferential targeting of L5 CP neurons over neighboring L3/5 CA neurons in IL cortex. We conclude by showing that BLA input is laminar-specific by producing stronger excitatory responses CA neurons in L3/5 compared with L2 in IL cortex. Collectively, this study reveals differential targeting of the BLA to PL and IL cortex, which depends both on laminar location and projection target of cortical neurons. Overall, our findings should have important implications for understanding the processing of pain and fear input by the PL and IL cortex.

## Significance Statement

The prelimbic (PL) and infralimbic (IL) cortices are comprised of a heterogeneous population of pyramidal neurons that receive, integrate, and relay information ascending from subcortical (long-range) origins. For the first time, we address critical unknowns regarding long-range targeting of the basolateral amygdala (BLA) to specific PL and IL neurons projecting to key subcortical structures involved in pain and fear, the periaqueductal gray (PAG), and the amygdala. Our data reveal a distinctive pattern of BLA input to these neurons demonstrating that BLA–mPFC connections are region-, laminar-, and projection-specific. Elucidating the organization and strength of long-range connections to specific neurons in the PL and IL cortex provides valuable insight into cortical processing of outputs relevant in pain and anxiety disorders.

## Introduction

The prelimbic (PL) and infralimbic (IL) cortex are adjacent regions shown to have distinct roles in integrating sensory and emotional information. A main focus of the PL–IL dichotomy has involved mainly fear behavior. Specifically, the PL cortex is implicated in fear expression (Vidal-Gonzalez et al., 2006; Corcoran and Quirk, 2007; Burgos-Robles et al., 2009; Laurent and Westbrook, 2009; Sierra-Mercado et al., 2011; Fitzgerald et al., 2014), whereas the IL cortex is critical for fear extinction (Milad and Quirk, 2002; Sierra-Mercado et al., 2006; Laurent and Westbrook, 2009; Chang and Maren, 2011; Sierra-Mercado et al., 2011; Holmes et al., 2012; Do-Monte et al., 2015). Distinct subpopulations of neurons in the basolateral amygdala (BLA) target the PL and IL cortex and display increased firing associated with fear conditioning and fear extinction, respectively (Senn et al., 2014). Long-range input from ascending BLA projections excites pyramidal neurons in layer 2 (L2) of the medial prefrontal cortex (mPFC; Little and Carter, 2012) preferentially targeting neurons with projections returning to the BLA (Little and Carter, 2013). However, the differential laminar targeting and connectivity of BLA inputs to specific PL and IL neurons identified by subcortical projection target has not been studied.

For this study, we were interested in dissecting BLA targeting of projectionally defined pyramidal neurons in L2 and L5 in both the PL and IL cortex. A major subcortical target of L5 neurons in the mPFC is the periaqueductal gray (PAG; Ferreira et al., 2015). The PAG is a key midbrain structure within a network responsible for regulating multiple physiological responses, most notably descending inhibition of ascending nociceptive input (Basbaum and Fields, 1978; Behbehani, 1995). Studies show that the BLA evokes excitatory responses in pyramidal neurons in L5 of the mPFC (Ishikawa and Nakamura, 2003; Orozco-Cabal et al., 2006). Therefore, BLA is poised to have significant effects on descending pain regulation via input onto L5 cortico-PAG (CP) neurons in the mPFC.

Another major projection target of the mPFC is the amygdala (Hurley et al., 1991; Brinley-Reed et al., 1995; McDonald, 1998). Previous studies show that PL and IL connect to different regions of the amygdala with PL targeting the basolateral nuclei (Vertes, 2004; Gabbott et al., 2005) and IL targeting GABAergic central nucleus and intercalated cells (McDonald et al., 1996; Berretta et al., 2005; Amir et al., 2011). This has offered one mechanistic explanation for PL–IL dichotomy in fear conditioning paradigms (Peters et al., 2009). Yet, differential targeting of the BLA to corticoamygdalar (CA) neurons in PL versus IL is likely another contributing factor to this dichotomy.

Here we aimed to determine: (1) whether the BLA makes long-range connections to L5 CP neurons in mPFC and (2) whether the synaptic connectivity of the BLA to CP and CA neurons was different between PL and IL cortices of the mPFC. Using optogenetics and retrograde labeling, we show that the BLA differentially targets CP and CA neurons in PL versus IL and that this targeting depends on laminar location and projection target of the output neuron.

## Materials and Methods

### Animals

Wild-type C57Bl/6J mice (Harlan Laboratories; total: *n* = 66) of either gender (35–46 d old) were used in accordance with the animal care and use guidelines of Indiana University, The University of Notre Dame, and the National Institutes of Health.

### Surgical procedure for intracranial injections

Mice were anesthetized with 1.5% isoflurane in 100% O_2_ with a flow rate of 0.6 L/min (SurgiVet Isotech 4, Smith). Body temperature was maintained at 37°C using a feedback-controlled heating pad. The head was mounted in a stereotaxic frame (900 series, Kopf Instruments) and buprenorphine (0.03 mg/kg) was injected subcutaneously prior to the surgical procedure. The top of the head was shaved and betadine was used to disinfect the area. A midline incision was made to the scalp to expose the skull. For PAG and BLA injections, the scalp was incised, a craniotomy was made, the dura was reflected, and pipettes were advanced to reach the stereotaxic coordinates of the desired target. Following surgery, meloxicam (0.25 mg/kg) was injected subcutaneously for pain relief during recovery.

### Retrograde tracer and adenoviral injections

Tracer and virus injections were performed using a Hamilton syringe connected to a UltraMicoPump 3 driven by a Micro 4 MicroSyringe Pump Controller (World Precision Instruments). Retrograde tracers were either cholera toxin β-subunits conjugated with AlexaFluor 647 dye (Life Technologies) or fluorescent red Retrobeads IX (Lumafluor). Submicroliter volumes (∼100–200 nl) of retrograde tracer were injected at a rate of 100 nl/min. For anterograde expression of channelrhodopsin-2 (ChR2) recombinant adeno-associated virus AAV1.CAG.ChR2-Venus.WPRE.SV40 (Petreanu et al., 2007, 2009) was injected (50 − 100 nl) at a rate of 100 nl/min. The virus was purchased through the UPenn Vector Core (Addgene 20071). Stereotaxic coordinates for PAG injections were as follows (in mm relative to bregma): 3.4 caudal, 0.5 lateral, and 2.8–3.2 deep at a 0° angle off the vertical plane. For BLA injections, coordinates were as follows (in mm relative to bregma): 0.73 caudal, 3.4 lateral, and 4.6 deep at a 4° angle off the vertical plane.

### Slice preparation

Brain slices were prepared as previously described (Sheets et al., 2011; Ferreira et al., 2015) at postnatal days 35–46 (ie, 14–21 d after AAV-ChR2 injections). Modified coronal brain sections (spine of the blade tilted rostrally 15–25°s; 300 μm thick) containing mPFC were made by vibratome-sectioning the brain (VT1200S, Leica) in ice-cold cutting solution [composed of the following (in mm): 110 choline chloride, 25 NaHCO_3_, 25 d-glucose, 11.6 sodium ascorbate, 7 MgSO_4_, 3.1 sodium pyruvate, 2.5 KCl, 1.25 NaH_2_PO_4_, and 0.5 CaCl_2_]. Slices were transferred to artificial CSF [ACSF; composed of the following (in mm): 127 NaCl, 25 NaHCO_3_, 25 d-glucose, 2.5 KCl, 1 MgCl_2_, 2 CaCl_2_, and 1.25 NaH_2_PO_4_, aerated with 95% O_2/_5% CO_2_] at 37°C for 30 min. Slices were subsequently incubated in ACSF at 22°C for at least 1 h prior to electrophysiological and optogenetic experiments.

### Fluorescence imaging and analysis

Acute cortical slices were visualized under coolLED optics (Scientifica) and fluorescence intensity analyses were performed using custom routines in MATLAB (MathWorks). Images were rotated to align the pia horizontally and regions of interest spanning the entire cortical thickness and containing labeled axons in the PL cortex and IL cortex were selected. The pixel intensities in these regions-of-interest were averaged along the rows, yielding a profile representing the average pixel intensities along the medial–lateral axis, showing the radial distribution of YFP fluorescence in the mPFC. Next, we performed background subtraction to reduce the autofluorescence signal, by fitting a polynomial to the nonfluorescent portions of the profile and subtracting a calculated background profile from the raw profile.

### Optogenetic and electrophysiological recordings

Briefly, slices were transferred to the recording chamber of an upright microscope (BX51, Olympus), and held in place with short pieces of flattened gold wire (0.813 mm diameter; Alfa Aesar). Fluorescently labeled neurons were visualized using coolLED optics (Scientifica). Pipettes for whole-cell recordings were fabricated from borosilicate capillaries with filaments (G150-F, Warner) using a horizontal puller (P-97, Sutter), and filled with intracellular solution composed of the following (in mm): 128 Cs-methanesulfonate, 10 HEPES, 1 EGTA, 4 MgCl_2_, 4 ATP, and 0.4 GTP, 10 phosphocreatine, 3 ascorbate, and 0.05 AlexaFluor 594 or 488 (Molecular Probes), pH 7.3. EGTA was included both to facilitate seal formation and to reduce cytosolic calcium elevations induced by the various stimulus protocols used in these studies. ACSF was used as the extracellular recording solution. Recordings were targeted to retrogradely labeled pyramidal neurons 60–100 µm deep in the slice. Pipette capacitance was compensated; series resistance was monitored but not compensated, and required to be <35 MΩ for inclusion in the dataset. Recordings were filtered at 4 kHz and digitized at 10 kHz. Slices were ideally used 1.5–3 h after preparation, but some were used up to 6 h after preparation. Recordings were performed at 34°C. The ACSF was refreshed every 2 h. The recording temperature was controlled by an in-line heating system (TC324B, Warner). In voltage clamp configuration, excitatory (glutamatergic) and inhibitory (GABAergic) responses during photoactivation of ChR2-positive BLA projections were recorded at command voltages of −70 mV and +10 mV, respectively. Wide-field photoactivation (40 mW, 3 ms) of ChR2-positive BLA axons was performed using a 470 nm wavelength LED (CoolLED pE excitation system) in line with a GFP filter (ET FITC/GFP, Olympus) and a 4× objective. For monosynaptic recordings, application of TTX (0.5 µm) and CPP (5 µm) was added to eliminate polysynaptic and NMDAR activity, respectively, and 4-AP (0.1 mm) was added to restore glutamate release (Petreanu et al., 2009; Little and Carter, 2012, 2013). Paired comparisons of monosynaptic strength were done in sequential recordings of labeled neurons within the same brain slice to limit variability due to AAV infection variability and/or slice orientation. The order of recorded neurons was alternated between slices.

### Glutamate uncaging and laser scanning photostimulation

Glutamate uncaging and laser scanning photostimulation (glu-LSPS) were performed using an ultraviolet (UV) laser (355 nm; DPSS Lasers). Stock solutions of MNI-caged glutamate (50 mm in water) were prepared at room temperature (to avoid precipitation) with sonication, aliquoted, and stored at −20°C until use. Ephus software was used for hardware control and data acquisition (http://www.ephus.org; Suter et al. 2010). The bath solution for photostimulation studies contained elevated concentrations of divalent cations (4 mm Ca^2+^ and 4 mm Mg^2+^) and an NMDA receptor antagonist (5 μm CPP; Tocris Bioscience), to dampen neuronal excitability. Caged glutamate (0.2 mm; MNI-glutamate, Tocris Bioscience) was added to the bath solution. Voltages were not corrected for liquid junction potential. Recordings were performed at 21°C and were monitored for series resistance (inclusion criterion: <40 MΩ; mean: ∼25 MΩ). For excitatory recordings, patch pipettes contained potassium-based intracellular solution (in mm: 128 K-gluconate, 10 HEPES, 1 EGTA, 4 MgCl_2_, 4 ATP, and 0.4 GTP, 10 phosphocreatine, 3 ascorbate, and 0.05 AlexaFluor 594 or 488 hydrazide). For inhibitory recordings, equimolar cesium was substituted for potassium, and 1 mm QX-314 was added to the intracellular solution. Photostimulation sites resulting in activation of glutamate receptors in the membrane of the recorded neuron were readily detected based on characteristically short onset latencies (<7 ms) of responses (Schubert et al., 2001; Anderson et al., 2010), and excluded from analysis. All remaining recorded inputs with onset latencies greater than 7 ms were included in the map analysis as synaptic responses resulting from uncaged glutamate activation of presynaptic neurons within the local circuit. Excitatory (glutamatergic) responses were recorded at a command voltage of −70 mV. Excitatory input maps (see Fig. 3) were constructed on the basis of the mean inward current over a 0–50 ms poststimulus time window. Inhibitory (GABAergic) responses were recorded at a command voltage of +10 mV. Inhibitory input maps were constructed on the basis of the mean outward current over a 0–750 ms poststimulus time window. Animal numbers reported for inhibitory maps are a subset of animals reported for excitatory maps because excitatory and inhibitory maps were acquired in the same recorded neuron. In some cases following acquisition of excitatory input maps, the recording was lost and subsequent inhibitory maps could not be acquired.

### Data analysis

Data acquisition and statistical analysis was performed using MATLAB routines (MathWorks). Data are represented as means with error bars showing the SEM. Group comparisons were performed with the Student’s *t* test (for normally distributed data) or the Wilcoxon rank sum test (for non-normally distributed data). Significance was set at *p* < 0.05. Superscript letters listed with *p*-values correspond to the statistical tests shown in Table 1.

**Table 1.** Statistical tests

	**Data**	**Test**	**Data**	**Power, %**	**Effect size**
a	Unknown	Paired *t* test	IL-CP excitatory	14.3	0.33
b	Normal	Paired *t* test	IL-CP inhibitory	97.3	1.62
c	Normal	Paired *t* test	PL-CP excitatory	77.9	0.97
d	Normal	Paired *t* test	PL-CP inhibitory	98.9	1.52
e	Normal	Unpaired *t* test	PL-CP vs IL-CP	91.8	1.45
f	Unknown	Paired *t* test	PL-CP vs IL-CP	77.2	1.03
g	Normal	Paired *t* test	PL-CA vs IL-CA	67.6	1.09
h	Unknown	Paired *t* test	IL-CP vs IL-CA	63.6	1.04
i	Normal	Paired *t* test	L2 vs L5 IL-CA	89.4	1.33

Data normality was determined using a Lilliefors test. Nonparametric statistical analysis produced similar results. *Post hoc* power analysis was performed using G*Power 3(v3.1.9.2, www.Gpower.hhu.de; Faul et al., 2007).

## Results

### Axons originating in the basolateral amygdala are differentially expressed in PL and IL regions of the mPFC

**Figure 1.**
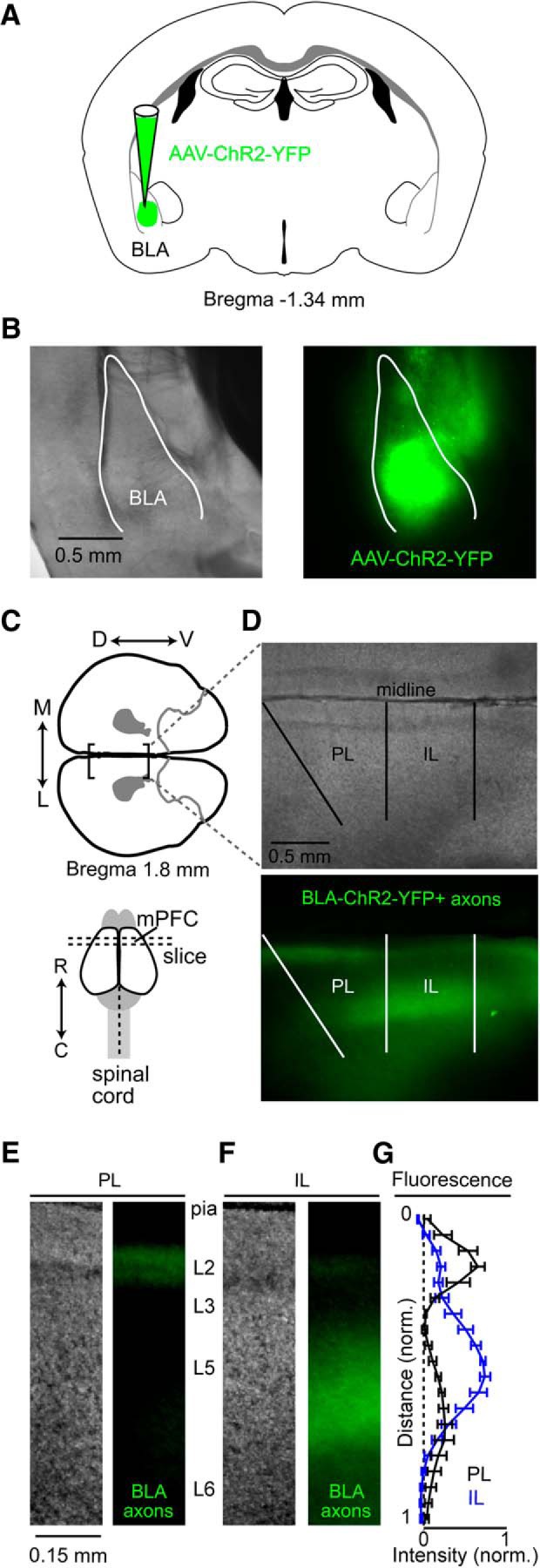
Expression of BLA axons in PL and IL regions of the mPFC. ***A***, Schematic of AAV-ChR2-YFP injection into the BLA. ***B***, A representative coronal brain section (left, bright-field; right, epifluorescence) showing AAV-ChR2-YFP injection into the BLA. ***C,*** Bottom, Schematic of the rostral-caudal (R ↔ C) location of coronal slice containing the mPFC. ***C***, Top, Schematic of a coronal brain section oriented so that the pia of both the PL and IL cortex was horizontal in the imaging chamber. ***D***, A representative image of a coronal brain section containing PL and IL cortex (top, bright-field; bottom, epifluorescence) in which BLA axons were anterogradely labeled with YFP from AAV-ChR2-YFP. ***E***, ***F***, Bright-field and epifluorescence images showing laminar distribution of BLA expression in PL and IL. ***G***, Normalized fluorescence intensity (mean ± SEM) of BLA axons (*n* = 5; 5 animals) as a function of normalized cortical distance (where pia = 0 and white matter = 1) in PL (black) and IL (blue). Dorsal-ventral, D↔V; lateral-medial, L↔M.

Injection of AAV-ChR2-YFP into the BLA ( Fig. 1*A*,*B*) resulted in robust labeling of YFP+ BLA axons in the medial prefrontal cortex (mPFC; Fig. 1*C*,*D*). However, the coronal expression pattern of BLA axons differed between the PL and IL regions of mPFC (Fig. 1*D*). Specifically, the fluorescence intensity of BLA axons in L2 was greater in PL compared to IL, whereas the expression in L5 was observed to be uniform across IL, but was limited in the PL cortex (Fig. 1*E*,*F*). This expression pattern indicates that BLA synaptically targets neurons based on both their regional and laminar location within the mPFC and hence could differentially affect local circuit activity within the PL versus the IL cortex.

### The BLA differentially targets CP neurons in the PL and IL cortex

**Figure 2.**
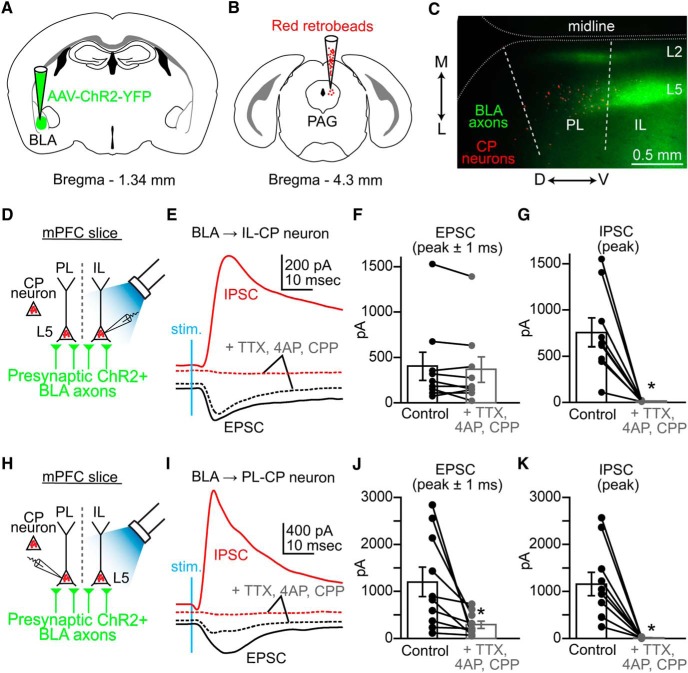
CP neurons in L5 of the PL and IL cortex receive monosynaptic and polysynaptic input from the BLA. ***A***, Injection of AAV-ChR2-YFP into the BLA. ***B***, Injection of retrograde tracer into the PAG. ***C***, Epifluorescence image overlay of brain slice showing regional and laminar distribution of YFP positive BLA axons (green) and red beads illuminated CP neurons (red) in the PL and IL cortical regions (outlined with dotted lines). Layers 2 and 5 are marked as L2 and L5, respectively (dorsal-ventral, D↔V; lateral-medial, L↔M). ***D***, Schematic showing how synaptic input was measured in whole-cell recordings of retrogradely labeled L5 CP neurons in IL cortex during wide-field activation of ChR2+ BLA axons via blue LED stimulation. ***E***, Examples of EPSC (black trace; holding at −70 mV) and IPSC (red trace; holding at +10 mV) recorded from L5 IL-CP neurons before and after addition of polysynaptic blockers (TTX 0.5 µm, 4-AP 0.1 mm, CPP 5 µm: dashed traces). ***F***, Paired comparison of recorded EPSC values in IL-CP neurons before (black) and after addition of polysynaptic blockers (gray). ***G***, Paired comparison of recorded IPSC values for IL-CP neurons before (black) and after addition of polysynaptic blockers (gray). ***H***, Schematic showing how synaptic input was measured in whole-cell recordings of retrogradely labeled L5 CP neurons in PL cortex during wide-field activation of ChR2+ BLA axons via blue LED stimulation. ***I***, Examples of a BLA evoked EPSC (black trace; holding at −70 mV) and IPSC (red trace; holding at +10 mV) recorded from L5 PL-CP neurons before and after addition of polysynaptic blockers (TTX 0.5 µm, 4-AP 0.1 mm, CPP 5 µm: dashed traces). ***J***, ***K***, Paired comparison showing a significant decrease in BLA evoked EPSCs and IPSCs recorded from PL-CP neurons after addition of polysynaptic blockers. * = p < 0.05; error bars = SEM.

To determine BLA connectivity to CP neurons in PL and IL cortex, we injected AAV-ChR2-YFP into BLA and retrograde tracer into the PAG (Fig. 2*A*,*B*). Labeled CP neurons were identified in L5 of PL and IL cortex, which overlapped with the expression of BLA axons (Fig. 2*C*). Wide-field stimulation of BLA axons via 470 nm LED light elicited both EPSCs and IPSCs in whole-cell recorded IL-CP neurons (*n* = 9 neurons/7 mice; Fig. 2*D*,*E*). We added TTX (0.5 µm) and CPP (5 µm) to eliminate polysynaptic and NMDAR activity, respectively, and added 4-AP (0.1 mm) to restore glutamate release. Recorded EPSCs (mean of peak ± 1 ms) in IL-CP neurons (401 ± 154 pA) decreased only marginally after treatment with blockers (365 ± 142 pA; *p* = 0.18^a^; Student’s paired *t* test) indicating that BLA axons primarily make monosynaptic excitatory connections onto AMPA receptors in IL-CP neurons (Fig. 2*E*,*F*). Recorded IPSCs (peak; 655 ± 71 pA) were eliminated by polysynaptic blockers (12.9 ± 1.5 pA; *p* = 0.001^b^; Student’s paired *t* test) confirming that BLA inhibition of IL-CP neurons is driven by activation of inhibitory interneurons within the local circuitry of the IL cortex (Fig. 2*G*). Stimulation of BLA axons also produced both EPSCs (1200 ± 314 pA) and IPSCs (1156 ± 249 pA) in PL-CP neurons (*n* = 10 neurons/7 mice; Fig. 2*H*,*I*). However, addition of polysynaptic blockers significantly reduced BLA-driven EPSCs (291 ± 76.8 pA; *p* = 0.02^c^) and IPSCs (15.3 ± 4.5 pA; *p* = 0.0002^d^) in PL-CP neurons (Fig. 2*I*–*K*). Remaining EPSC after blockers suggested that BLA input to PL-CP neurons consists of both polysynaptic and monosynaptic connections.

**Figure 3.**
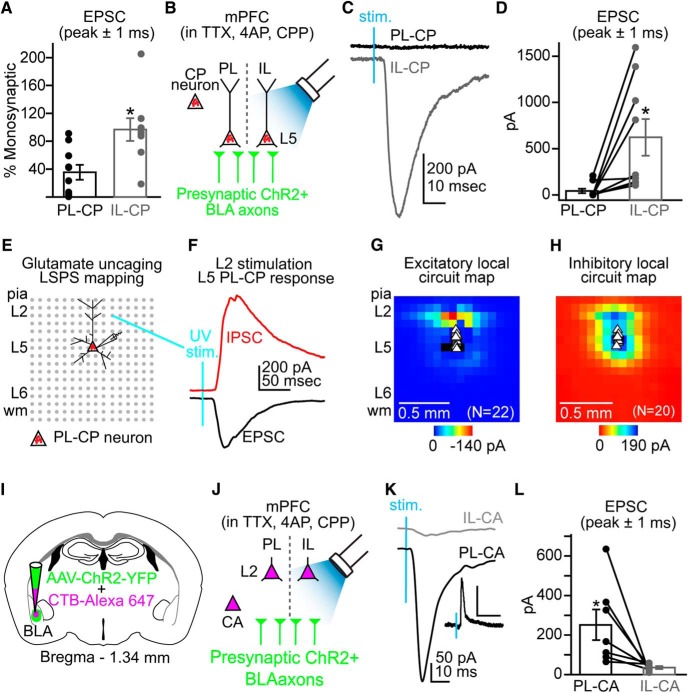
The BLA differentially targets CP neurons in PL and IL cortex. ***A***, Comparison of the percentage EPSC (peak ± 1 ms) remaining following application of polysynaptic blockers for PL-CP and IL-CP neurons. ***B***, Experimental paradigm for comparing monosynaptic input (in TTX, 4AP, CPP) to retrogradely labeled L5 CP neurons in PL and IL during wide-field activation of ChR2+ BLA axons via blue LED stimulation. ***C***, Examples of monosynaptic EPSCs for a PL-CP neuron (black trace) and IL-CP neuron (gray trace) in the same slice following blue LED stimulation (stim) of ChR2+ BLA axons. ***D***, Paired comparison of EPSC values for PL-CP and IL-CP neurons. ***E***, Schematic representation for the 16 × 16 stimulation grid (75 µm spacing) using for local circuit mapping of retrogradely labeled PL-CP neurons using UV-assisted glutamate uncaging (L, layer; wm, white matter). ***F***, Examples of an EPSC (black trace; holding at −70 mV) and an IPSC (red trace; holding at +10 mV) recorded from L5 PL-CP neurons following UV activation of caged glutamate in L2 of the PL cortex. ***G***, Average of excitatory local circuit maps showing robust L2 input to PL-CP neurons. ***H***, Average of excitatory inhibitory circuit maps showing robust inhibitory input to PL-CP neurons following activation of both L2 and L5. ***I***, Coinjection of AAV-ChR2-YFP and CTB-AlexaFluor 647 (cholera toxin β-subunits conjugated with AlexaFluor 647) into the BLA. ***J***, Experimental paradigm for comparing monosynaptic input to retrogradely labeled L2 PL-CA and L2 IL-CA neurons during wide-field activation of ChR2+ BLA axons via blue LED stimulation. ***K***, Examples of monosynaptic EPSCs for a L2 IL-CA neuron (gray trace) and a L2 PL-CA neuron (black trace) in the same slice following blue LED stimulation (stim) of ChR2+ BLA axons. ***K*,** Inset, Action potential firing in response to blue LED stimulation recorded in cell-attached mode from a L2 PL-CA neuron. Scale bars: 5 mV, 20 ms. ***L***, Paired comparison of EPSC values for L2 IL-CA and L2 PL-CA neurons. *p < 0.05; error bars = SEM.

Overall, we found that percent EPSC remaining (mean of peak ± 1 ms) following addition of polysynaptic blockers was significantly lower for PL-CP neurons (35.4 ± 10.6%) compared with L5 IL-CP neurons (96.7 ± 16.4%; *p* = 0.005; Student’s unpaired *t* test; Fig. 3*A*). Based on this finding, we hypothesized that excitatory monosynaptic input originating in the BLA was stronger to IL-CP neurons compared to PL-CP neurons. In the presence of polysynaptic blockers, we performed sequential recordings from pairs of L5 PL-CP and IL-CP neurons in the same brain slice and measured EPSCs elicited by optogenetic activation of BLA axons (Fig. 3*B*). We found that BLA input evoked significantly larger monosynaptic EPSCs in IL-CP neurons (622 ± 119 pA) compared to PL-CP neurons (44.5 ± 26.8 pA; *n* = 9 pairs/7 mice; *p* = 0.016^f^; Student’s paired *t* test Fig. 3*C*,*D*). These data demonstrated preferential monosynaptic targeting of BLA input to L5 CP neurons in IL versus PL cortex.

Because wide-field excitation of BLA inputs elicited polysynaptic responses in PL-CP neurons, we used glu-LSPS to map the local excitatory and inhibitory circuits for PL-CP neurons. Glutamate was “uncaged” via UV stimulation (20 mW, 1 ms) in a 16 × 16 grid (75 µm spacing) tangent with the pia and centered horizontally with the PL-CP soma (Fig. 3*E*). As previously described (Ferreira et al., 2015), responses were recorded in voltage-clamp mode (−70 mV: excitatory; +10 mV: inhibitory; Fig. 3*F*) to produce a trace map of input from local presynaptic locations which we converted to color maps for visualization. Input maps (*n* = 22 neurons/14 mice) showed that PL-CP neurons receive strong local excitatory and inhibitory input from L2 (Fig. 3*F*–*H*). Local inhibitory maps also show robust IPSCs in PL-CP neurons following perisomatic activation of L5 neurons (Fig. 3*H*). It has been previously shown that IL-CP neurons also receive strong local excitatory and inhibitory input from L2 (Ferreira et al., 2015). However, BLA axon expression is low in L2 of IL (Fig. 1*D*) and stimulation of BLA input produced mainly monosynaptic EPSCs in IL-CP neurons (Fig. 2*F*). This suggests that BLA evoked responses in IL-CP neurons does not involve the activation of a local L2→L5 pathway.

We postulated that the strong polysynaptic and monosynaptic input to PL-CP and IL-CP neurons, respectively, is, at least in part, due to differences in the targeting of the BLA to L2 in PL and IL cortex. A major class of L2 output neurons in the mPFC is CA neurons (Little and Carter, 2013; Ferreira et al., 2015). We next coinjected retrograde tracer and AAV-ChR2-YFP into the BLA and measured the strength of monosynaptic BLA input in sequential recordings of L2 PL-CA and IL-CA neurons (Fig. 3*I*,*J*). Activation of BLA input evoked significantly larger EPSCs (mean of peak ± 1 ms) in L2 PL-CA neurons (251.6 ± 78 pA) compared with L2 IL-CA neurons (35.4 ± 6.5 pA; *n* = 7 pairs/6 mice; *p* = 0.04^g^; Fig. 3*K*,*L*).This is consistent with the stronger BLA axonal expression seen in L2 of PL cortex over IL cortex (Fig. 1). Previous work has shown that the BLA can drive action potential firing of CA neurons in L2 of the mPFC (Little and Carter, 2013). Given the robust BLA axon expression in L2 of PL, we tested whether optogenetic stimulation of BLA inputs was sufficient to drive firing in retrogradely labeled L2 CA neurons in PL. Performing cell-attached recordings in current-clamp configuration, we found that LED stimulation (3 ms) of BLA axons could elicit firing of L2 PL-CA neurons (Fig. 3*K*, inset). Based on our local mapping results, this finding suggests that BLA input to PL-CP neurons may involve local processing through L2 CA neurons.

### Projection identity and laminar location of mPFC neurons determines strength of BLA monosynaptic input

**Figure 4.**
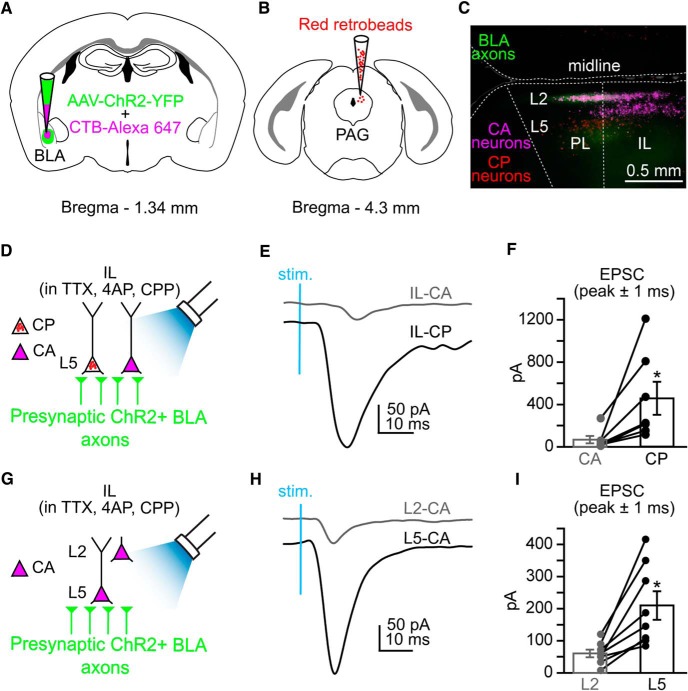
Targeting of BLA axons in the IL cortex is projection and laminar specific. ***A***, Coinjection of AAV-ChR2-YFP and CTB-AlexaFluor 647 into the BLA. ***B***, Injection of red Retrobeads IX into the PAG. ***C***, Epifluorescence image overlay of the mPFC showing regional and laminar distribution of CA neurons (purple), BLA axons (green), and CP neurons (red) in the PL and IL cortical regions of the mPFC. ***D***, Experimental paradigm for comparing monosynaptic input to L5 CP and CA neurons in IL during wide-field activation of ChR2+ BLA axons via blue LED stimulation. ***E***, Examples of monosynaptic EPSCs for a L5 IL-CP neuron (gray trace) and a L5 IL-CA neuron (black trace) in the same slice following blue LED stimulation (stim) of ChR2+ BLA axons. ***F***, Paired comparison of monosynaptic EPSC values for L5 IL-CA and L5 IL-CP neurons. ***G***, Experimental paradigm for comparing monosynaptic input to retrogradely labeled L2 and L5 CA neurons in IL during wide-field activation of ChR2+ BLA axons via blue LED stimulation. ***H***, Examples of monosynaptic EPSCs for a L2 IL-CA neuron (black trace) and a L5 IL-CA neuron (gray trace) in the same slice following blue LED stimulation (stim.) of ChR2+ BLA axons. ***I***, Paired comparison of monosynaptic EPSC values for L2 IL-CA and L5 IL-CA neurons. * = p < 0.05; error bars = SEM.

Preferential synaptic connectivity of the BLA onto specific L2 cell types in the mPFC has been shown previously (Little and Carter, 2013). In addition to L2, CA neurons are distributed in L3 and L5 of IL cortex (Ferreira et al., 2015). We hypothesized that cell-type-specific targeting of the BLA also occurred between CA and CP neurons in L5 of IL cortex. We injected AAV-ChR2-YFP into the BLA and used fluorescently distinct retrograde tracers to identify IL-CP and IL-CA neurons in the same slice (Fig. 4*A–C*). Performing sequential whole-cell recordings from pairs of neighboring L5 IL-CP and L3/5 IL-CA neurons (Fig. 4*D*), we found that BLA inputs evoked significantly larger EPSCs (mean of peak ± 1 ms) in L5 IL-CP neurons (458 ± 156 pA) compared with L3/5 IL-CA neurons (67.0 ± 35 pA; *n* = 7 pairs/8 mice; *p* = 0.03^h^; Student’s paired *t* test; Fig. 4*E*,*F*). These data support our hypothesis by showing that the BLA preferentially targets L5 CP neurons over L3/5 CA neurons in IL cortex.

Because CA neurons are distributed in L2 and L3/5 in IL cortex (Fig. 4*C*) and because the labeling pattern of BLA axons in mPFC showed robust expression in L5 compared with L2 in IL cortex (Fig. 1), we hypothesized that BLA input would be stronger in L3/5 IL-CA neurons compared with L2 IL-CA neurons (Fig. 4*G*). Again in the presence of polysynaptic blockers, sequential whole-cell recordings revealed that BLA inputs evoked significantly larger EPSCs (mean of peak ± 1 ms) in L3/5 IL-CA neurons (210 ± 44 pA) compared with L2 IL-CA neurons (60.5 ± 12 pA; *n* = 8 pairs/7 mice; *p* = 0.005^i^; Fig. 4*H*,*I*). These data show that the strength of BLA input to CA neurons in IL cortex depends on laminar location of the soma.

## Discussion

Our findings reveal unknown details regarding synaptic targeting of the BLA onto pyramidal neurons in the mPFC relevant in the endogenous analgesic network and in neural pathways relevant in fear conditioning. We combined retrograde tracing, brain-slice electrophysiology, and viral optogenetics to delineate the long-range targeting of the BLA onto defined CP and CA neurons in the mouse PL and IL cortex. We show that the BLA preferentially targets L5 CP neurons in IL cortex over the PL cortex. We also show that monosynaptic inputs originating in the BLA are stronger to L5 CP neurons than neighboring L3/5 CA neurons in IL cortex. This type of preferential connectivity of BLA input has been demonstrated in L2 of the mouse mPFC (Little and Carter, 2013). However, this previous study showed that excitatory inputs from the BLA were stronger to L2 CA neurons compared to neighboring neurons projecting to the contralateral mPFC. Our results complement these findings and also demonstrate that the BLA preferentially and monosynaptically targets pyramidal neurons in L5 of IL cortex based on subcerebral projection target.

**Figure 5.**
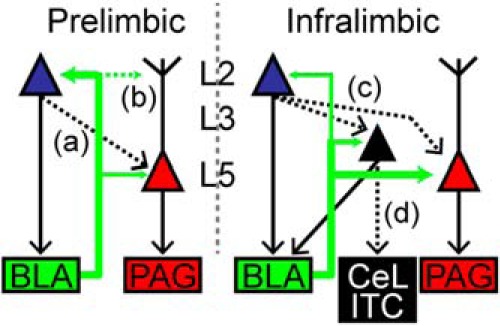
Working circuit diagram for BLA targeting of CP and CA neurons in PL and IL cortex. In PL cortex, the BLA sends robust projections to L2 PL-CA and weaker projections L5 PL-CP neurons. A local L2 CA → L5 CP pathway (***a***) and targeting of the BLA to apical dendrites of L5 PL-CP neurons (***b***) remain unresolved. In IL cortex, the BLA preferentially targets L5 IL-CP neurons over L3/5 IL-CA neurons. The BLA also preferentially targets L3/5 IL-CA neurons over L2 IL-CA neurons. Local L2 CA → L5 CP and L2 CA → L3/5 CA pathways (***c***) and targeting of L3/5 CA neurons specifically to the central lateral amygdala (CeL) and intercalated (ITC) regions (***d***) remain unresolved. Thickness of green arrows = relative strength of connection; dashed lines = unresolved connections.

Polysynaptic excitatory responses in L5 PL-CP neurons following stimulation of BLA projections indicated activation of local cortical pathways in PL cortex. Dissection of local circuit pathways has uncovered a strong L2/3 → L5 projection across multiple cortical areas (Kampa et al., 2006; Weiler et al., 2008; Anderson et al., 2010; Hooks et al., 2011; Ferreira et al., 2015). Using laser-scanning photostimulation of caged glutamate, we show that L5 PL-CP neurons receive strong descending input from L2. Given that the preferential targeting of BLA to L2 PL-CA neurons can induce action potential firing, we speculate that polysynaptic activity recorded in PL-CP neurons during excitation of BLA axons may be due in part to activation of a L2 CA → L5 CP pathway (Fig. 5). Paired recordings of retrogradely labeled L2 CA and L5 CP will be necessary to confirm this speculation and will be one focus of future studies. Additionally, the interactions of the PL and IL cortex remain unclear. Optogenetic activation of IL pyramidal neurons can inhibit PL pyramidal neurons (Ji and Neugebauer, 2012). However, the laminar pathways of this interaction are unknown. It is conceivable that activation of L2 neurons in one cortical region (eg, L2 PL-CA) evokes feedforward inhibition of L5 neurons in the neighboring cortical region (eg, L5 IL-CP neurons). Detailed local circuit mapping is necessary to confirm this type of intercortical interaction.

Comprehensive retrograde tracing experiments suggest that the rat mPFC is divided into functionally overlapping yet distinct dorsal and ventral domains containing strategically organized pyramidal neurons that project to specific sets of other subcortical structures including the parabrachial nucleus, rostral ventral medulla, hypothalamus, and spinal cord (Gabbott et al., 2005). Given that these projection neurons are distributed primarily in L5 of both PL and IL cortex (Gabbott et al., 2005), our data suggest that the BLA will preferentially target those neurons in L5 of IL over PL cortex. However, targeting of BLA axons to apical dendrites of L5 pyramidal neurons in PL cortex still must be explored. Additional studies also provide evidence that functional differences occur dorsoventrally in the mPFC (Giustino and Maren, 2015). Our data clearly show that the BLA targets CP neurons, CA neurons, and inhibitory interneurons [likely parvalbumin-positive (PV+); Gabbott et al., 2006] in both PL and IL. However, our findings suggest that differences in laminar targeting and strength of inputs to these cortical neurons play a significant role in the functional dichotomy between these mPFC regions. We speculate that this holds true for other L2 and L5 projection neurons and inhibitory interneurons spanning both PL and IL. How these inputs are affected in nociceptive/antinociceptive or fear expression/extinction context requires further investigation.

The connectivity of ascending BLA inputs to L5 CP neurons in the mPFC has functional implications related to nociception. Stimulation of the mPFC can produce analgesia in rats (Hardy, 1985) and can alter the firing rate of PAG neurons responsive to noxious input (Hardy and Haigler, 1985). Ascending nociception activates the amygdala via the parabrachial area (Bernard et al., 1996; Gauriau and Bernard, 2002) and the thalamus (Lanuza et al., 2008). Further, lesions in the amygdala disrupt the conditional hypoalgesia that is driven by endogenous anti-nociceptive circuitry during a Pavlovian conditional stimulus (Helmstetter, 1992; Helmstetter and Bellgowan, 1993). Based on these findings, we speculate that BLA targeting of L5 CP neurons is a critical component of the BLA driven activity within the endogenous analgesic network recruited during painful stimuli and fear-conditioned analgesia. Additionally, persistent pain alters the BLA-mPFC pathway. In a rat model of arthritic pain, increased plasticity in the BLA contributes to both sensory and affective pain behavior and to increased polysynaptic inhibition of L5 neurons in PL cortex (Ji et al., 2010). Anatomical studies show that the BLA targets PV+ inhibitory neurons in rat mPFC, which likely contribute to “feedforward” inhibition of pyramidal neurons (Gabbott et al., 2006). Our results indicate that increased polysynaptic inhibition of L5 neurons in PL cortex would decrease output to the PAG potentially diminishing the suppression of ascending nociceptive signaling. Studies confirm that enhancing neurotransmission primarily in the PL cortex reduces neuropathic pain behavior (Millecamps et al., 2007; Lee et al., 2015), which may in part involve increased output to the PAG and the BLA.

In addition to altering the dynamics of cortical pain control, differential targeting of the PL and IL cortex by the BLA have implications in how mPFC networks affect fear conditioning. It has been shown that PL stimulation enhances fear expression and IL activity is critical for fear extinction (Milad and Quirk, 2002; Vidal-Gonzalez et al., 2006; Quirk and Mueller, 2008; Bukalo et al., 2015). Two distinct pathways originating in the BLA play a major role in the balance of activity between the PL and the IL during fear expression and fear extinction, respectively (Senn et al., 2014). Our findings indicate that increased BLA input to the IL detected during fear extinction (Senn et al., 2014) directly affects the activity of L5 IL-CP neurons, which target the ventral PAG (Ferreira et al., 2015). There is evidence suggesting that mPFC activity triggers opioid release within the PAG (Tracey and Mantyh, 2007; Wagner et al., 2007). Therefore, we suspect that enhanced signaling of the BLA to the IL will directly increase the activity of IL-CP neurons potentially leading to opioid release in the ventral PAG which is essential for fear extinction (McNally et al., 2004; McNally et al., 2011).

We also show that the BLA preferentially targets CP neurons over CA neurons in L5 of IL cortex. Nonetheless, the BLA does directly excite CA neurons in IL cortex preferentially targeting CA neurons in L3/5 over L2. Previous studies propose that CA neurons in IL cortex target and activate the GABAergic cells (ITC) in the amygdala, which are involved in the retrieval of fear extinction memories (Quirk et al., 2003; Likhtik et al., 2008; Amir et al., 2011). However, new evidence suggests that IL activation of the ITC occurs indirectly through the stimulation of the BLA (Strobel et al., 2015). Another study showed that in addition to targeting the BLA and ITC, IL cortex sends robust projections to the central lateral amygdala (CeL; Pinard et al., 2012). The CeL inhibits output neurons of central medial amygdala (CeM; Ciocchi et al., 2010) including neurons projecting to the PAG (Haubensak et al., 2010), which are important for initiating fear responses (Paré et al., 2004). Indeed, fear extinction involves IL-mediated inhibition of CeM output (Amano et al., 2010), which may be driven via activation of ITC and/or CeL pathways.

A subpopulation of neurons projecting to the perirhinal cortex (PRC) is found in both L2/3 and L5 of the rat frontal cortex (Hirai et al., 2012). However, it was found that these PRC-projecting L2/3 and L5 neurons displayed different firing properties and targeted distinct areas within the PRC (Hirai et al., 2012). This suggests that subsets of cortical neurons projecting to different subregions within cortical and subcortical structures have distinct laminar and electrophysiological profiles. In mouse mPFC, L2 IL-CA and L3/5 IL-CA neurons have distinct subthreshold properties (Ferreira et al., 2015) and therefore may target specific subregions within the amygdala. It has been shown that L2 IL-CA and L3/5 IL-CA are intrinsically different (Ferreira et al., 2015) and therefore may have distinct projection targets within the amygdala. We speculate that L2 IL-CA neurons may project mainly to the BLA, whereas L3/5 IL-CA neurons target the CeL and ITC (Fig. 5). It is conceivable that input from L2 IL-CA neurons to BLA would inhibit CeM output via activation of ITC (Amano et al., 2010) or through excitation of GABAergic CeL neurons (Tye et al., 2011). However, it is also possible the L3/5 IL-CA neurons target PKC-δ+ CeL neurons which inhibit CeM output to the PAG (Haubensak et al., 2010) thereby decreasing fear response. Other evidence shows that PKC-δ− CeL neurons send direct inhibitory input to the PAG and that synaptic activity of these neurons is increased by fear conditioning (Penzo et al., 2014). Because PKC-δ+ neurons inhibit PKC-δ− (Haubensak et al., 2010) activation of PKC-δ+ neurons by IL inputs may decrease PKC-δ− driven inhibition of the PAG. Therefore, the increased BLA input to the IL cortex observed during fear extinction (Senn et al., 2014) likely involves eliciting output of L5 IL-CP neurons, which directly drive activity of the PAG in combination with eliciting output of L2 and L3/5 IL-CA neurons, which indirectly disinhibits the PAG via CeL and ITC pathways (Fig. 5). An intriguing future direction will involve experiments testing the local connectivity between CP and CA neurons in IL cortex by using paired recordings and/or optogenetic techniques (Fig. 5).

The PL cortex is critical for fear expression and the reduction of extinction retrieval (Vidal-Gonzalez et al., 2006; Corcoran and Quirk, 2007; Burgos-Robles et al., 2009; Sotres-Bayon et al., 2012). We found that the BLA evokes stronger excitatory input to L2 CA neurons in PL over IL cortex. Output of the PL to the amygdala (ie, L2 PL-CA neurons) consists of excitatory projections (Brinley-Reed et al., 1995) targeting the BLA (Vertes, 2004; Gabbott et al., 2005). The BLA sends excitatory input to the CeL (Samson et al., 2005), which disinhibits CeM output and leads to conditioned fear responses (Ciocchi et al., 2010). Yet, a more recent study shows that the BLA-CeL pathway reduces CeM output leading to anxiolytic behavioral effects (Tye et al., 2011). However, this same study showed that a subpopulation of BLA neurons can directly excite the CeM, which contributes to increased anxiety behavior (Tye et al., 2011). It is possible that BLA neurons responsible for increased activation of the PL cortex during states of high fear (Senn et al., 2014) concurrently excite CeM neurons. Moreover, BLA targeting of PL cortex involves a strong and excitatory reciprocal signal back from L2 neurons, which may target BLA neurons responsible for direct enhancement of CeM output. In addition to sending input back to the BLA, it is likely that L2 PL-CA neurons also alter output to the PAG through local descending connections onto L5 PL-CP neurons (Fig. 5). This level of local processing for coordinating descending input to the BLA and PAG is a possible mechanism that distinguishes function of PL and IL cortex.

Collectively, this study shows that the BLA differentially targets PL and IL cortical output to the PAG and amygdala (Fig. 5). Because the PAG and amygdala are subcortical structures involved in regulating multiple physiological responses, most notably pain, anxiety, and autonomic regulation, we anticipate our findings will offer new insight into differential roles for PL and IL cortex in pain and anxiety disorders.

## Synthesis

The decision was a result of the Reviewing Editor Menno Witter and the peer reviewers coming together and discussing their recommendations until a consensus was reached. A fact-based synthesis statement explaining their decision and outlining what is needed to prepare a revision is listed below. The following reviewers agreed to reveal their identity: Stephen Maren, Gregory Quirk

This is a very nicely done study that dissects the neural circuitry between the BLA and subregions within mPFC by examining the synaptic physiology and targets of basolateral amygdala (BLA) projections to the prefrontal cortex (PFC). While it is known that mPFC receives monosynaptic input from the BLA, little is known about the interactions between BLA inputs and its specific mPFC targets. Indeed, little is known about sources/targets of mPFC inputs in general. The authors address this gap in knowledge by combining optogenetics and glutamate uncaging in a slice recording preparation to differentiate BLA projections onto either PL or IL with laminar specificity. Emphasis is placed on the differential targeting of long-range projection neurons in the prelimbic (PL) and infralimbic (IL) divisions of the PFC by BLA neurons. BLA neurons project more heavily onto periaqueductal projecting neurons in IL layer 5, whereas they project strongly to neurons in PL layer 2 that project to BLA, as well as target PAG polysynaptically via Layer 5.. This interesting pattern of projection targets sheds new light on the role of amygdala-PFC projections in pain regulation and extinction learning. This important work will be useful to many scientists mapping the function of these circuits in these behavioral processes. And, although there is clearly considerable evidence that PL and IL perform dichotomous functions, this is not the rule.

These findings provide (for the first time) evidence for a circuit for fear conditioned hypoalgesia, a well-established conditioned response. Provides new details on amygdala-prefrontal synaptic connectivity in the context of long -range cortical-PAG and cortical-amygdala circuits and connects BLA anatomy with growing literature on PL IL differences. The findings also suggest that BLA inputs to IL could facilitate extinction, while BLA inputs to PL could facilitate fear expression. The laminar differences between BLA targets in PL and IL open new questions about the organization of other mPFC inputs, cell types, and microcircuitry of pain and conditioned fear.

There are only a few minor suggestions to improve the clarity of this manuscript:

1. The authors show a clear and segregated laminar distribution of fiber labeling in PL and IL (Figure 1D). Interestingly, these results mirror those observed in Gabbott et al., 2005, which suggested a difference in connectivity in ventral PL versus dorsal PL (see figures 14 and 16). The authors could cite and incorporate the Gabbott et al., 2005 study into the discussion.

2. It would be helpful if the authors added a circuit diagram to summarize their findings.

3. In the discussion, the authors could speculate about interactions between PL and IL. Perhaps Layer 2 PL neurons project to Layer 5 IL neurons, which may also influence pain perception and/or fear extinction. Given that they suggest different effects of PL and IL projections to BLA, they may want to speculate on different BLA targets of these projections. The authors might further spend some time in their discussion considering the shared aspects of BLA projections to PL and IL (see Giustino and Maren, 2015 for a recent review of PFC function in fear and extinction).
